# What decides the suspicion of acute coronary syndrome in acute chest pain patients?

**DOI:** 10.1186/1471-227X-14-9

**Published:** 2014-04-17

**Authors:** Alexander Kamali, Martin Söderholm, Ulf Ekelund

**Affiliations:** 1 Department of Otolaryngology-Head and Neck Surgery, Halmstad Hospital, Halland, Sweden; 2 Section of Emergency Medicine, Department of Clinical Sciences at Lund, Lund University, Lund, Sweden

## Abstract

**Background:**

Physicians assessing chest pain patients in the emergency department (ED) base the likelihood of acute coronary syndrome (ACS) mainly on ECG, symptom history and blood markers of myocardial injury. Among these, the ECG has been stated to be the most important diagnostic tool. We aimed to analyze the relative contributions of these three diagnostic modalities to the ED physicians’ evaluation of ACS likelihood in clinical practice.

**Methods:**

1151 consecutive ED chest pain patients were prospectively included. The ED physician’s subjective assessment of the patient’s likelihood of ACS (obvious ACS, strong, vague or no suspicion of ACS), the symptoms and the ECG were recorded on a special form. The ED TnT value was retrieved from the medical records. Frequency tables and logistic regression models were used to evaluate the contributions of the diagnostic tests to the level of ACS suspicion.

**Results:**

Symptoms determined whether the physician had any suspicion of ACS (odds ratio, OR 526 for symptoms typical compared to not suspicious of ACS) since neither ECG nor TnT contributed significantly (ORs not significantly different from 1) to this assessment. ACS was suspected in only one in ten patients with symptoms not suspicious of ACS. Symptoms were also more important (OR 620 for typical symptoms) than ECG (OR 31 for ischemic ECG) and TnT (OR 3.4 for a positive TnT) for the assessment of obvious ACS/strong suspicion versus vague/no suspicion. Of the patients with ST-elevation on ECG, 71% were considered to have an obvious ACS, as opposed to only 6% of those with symptoms typical of ACS and 10% of those with a positive TnT.

**Conclusion:**

The ED physicians used symptoms as the most important assessment tool and applied primarily the symptoms to determine the level of ACS suspicion and to rule out ACS. The ECG was primarily used to rule in ACS. The TnT level played a minor role for the assessment of ACS likelihood. Further studies regarding ACS prediction based on symptoms may help improve decision-making in ED patients with possible ACS.

## Background

Unstable angina pectoris (UA) or acute myocardial infarction (AMI), i.e. acute coronary syndrome (ACS) is one of the main killers in the western world. In Sweden (population 9.5 million), chest pain with possible ACS is one of the leading causes of emergency care, with an estimated 180,000 patients presenting to emergency departments (EDs) every year [1,2]. The treatment of ACS has improved dramatically over the last decades, but the diagnostic evaluation in the ED of patients with suspected ACS has been almost unchanged. This evaluation thus remains difficult, especially in the face of an ageing patient population with diverse symptoms and frequent comorbidities. Since clear diagnostic findings to rule ACS in or out are often lacking, patient management in the ED is normally based on the level of suspicion of ACS, i.e. the physician’s assessment of the patient’s likelihood of ACS.

Although the patient’s age, sex and the physical exam may play a role, the main tools to determine the likelihood of ACS are the symptom history, the ECG and blood markers of myocardial injury such as troponin T (TnT). The strengths, weaknesses and predictive values of these three diagnostic modalities have been extensively studied [3-19], and their theoretical importances analyzed. Based on these studies, the ECG has been stated to be the most valuable test [4,5]. It is still unclear however, just how these three diagnostic tools are used by ED physicians in their clinical reasoning, and which of them is the most important when physicians decide the likelihood of ACS.

This study aimed to analyze, in routine ED care, the relative contributions of the symptoms, ECG and TnT to the physician’s assessment of the patient’s overall likelihood of ACS.

## Methods

### Setting

The Skåne University Hospital at Lund is a 900 bed institution which serves as the primary hospital for some 290,000 inhabitants and has a cardiac intensive care unit with 19 beds. Percutaneous coronary intervention and coronary bypass surgery are available 24 hours a day. There is a traditional ED with approximately 65000 patients per year with physician interns, residents and specialists in internal and emergency medicine. During the study period, there were no standardized management protocols for patients with possible ACS, and no dedicated chest pain unit. Standard practice was however to admit patients at low risk to telemetry at the intermediate care ward, and to admit those at high risk to the cardiac intensive care unit. A prehospital ECG system was in operation with ambulance ECGs sent to a cardiologist on call. If an ST elevation myocardial infarction was identified, the patient was transported directly to the angiography laboratory, bypassing the ED.

### Patient inclusion and exclusion

**Figure 1:**
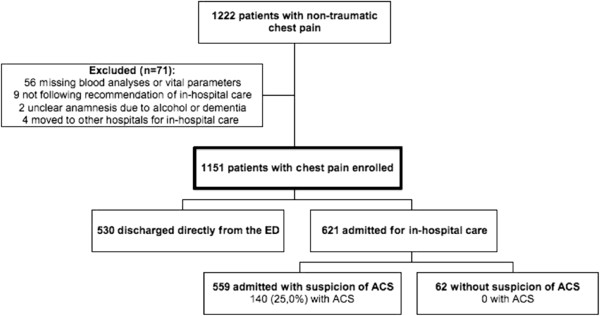
Patient flow chart.

All patients aged over 18 years presenting with non-traumatic chest pain as the chief complaint to the Lund ED at Skåne University Hospital between June 12th and October 8th 2009 were prospectively screened for the study, and patients were included if the physician’s assessment verified that the patient’s chief compliant was chest pain. Ongoing chest pain was not required for inclusion. Patients not following the physician’s recommendation of in-hospital care were excluded, as were patients unable to give a clear symptom history due to e.g. alcohol intoxication or dementia, and those transferred to other hospitals for in-patient care. Patient numbers and causes of exclusion are shown in Figure 1. All included patients underwent a routine clinical evaluation in the ED including symptom history, physical exam, ECG and TnT.

All included patients gave informed consent, and the study was approved by the regional ethics committee in Lund (DNR2009/630).

### Patient data collection

Shortly after the patient encounter, the responsible emergency physician made subjective assessments of the patient’s likelihood of ACS based on the symptoms, the ECG, and on the entire clinical picture. The assessments were noted on a study form either by the physician or by one of the authors (MS). During the assessments, all initial clinical data were available, including the first TnT. There was no retrospective review for quality or accuracy of the physicians’ assessments in the study; they were used “as is”. The troponin T (TnT) result available to the ED physician was retrieved from the electronic patient records. During the study period, values ≥ 0.05 μg/L were being considered indicative of ACS [20].

Symptoms reported by the patients were classified by the physician on the form according to a predefined scale as a.) typical of AMI or b.) typical of UA, c.) not specific for ACS or d.) not suspicious of ACS. The definitions of symptoms typical of AMI and UA were those generally used at the hospital during the study period, and followed the recommendations by the European Society of Cardiology, the American College of Cardiology and the American Heart Association [21,22]. These definitions were not provided on the study form. Where the physician noted two different degrees of suspicion, the strongest suspicion was registered for the study.

ECG changes recorded by the ED physicians were those defined in previous studies on risk scores in the ED [23-25]: ST-elevation or depression ≥ 1 mm in at least two anatomically contiguous leads, pathological Q-waves (>0,04 seconds and/or > 1/3 of the R-wave amplitude), T-wave inversion ≥ 1 mm in at least two leads. The physicians also recorded the presence of left bundle branch block (LBBB), atrial flutter (AFL) or fibrillation (AF) according to standard diagnostic criteria. In the present study, a normal ECG was defined as an ECG lacking all of the findings above. An ischemic ECG was defined as an ECG with ST elevation or depression or T-wave inversion. If the physician noted more than one ECG finding, the one indicating the highest risk of ACS (ST elevation > ST depression > T inversion > Q wave) was registered for the study.

The physician classified his or her overall level of ACS suspicion, i.e. the assessment of the patient’s likelihood of ACS based on the entire clinical picture, as obvious ACS, strong, vague or no suspicion of ACS. In order to limit heterogeneity of the physician assessments, suggested definitions of the different levels of suspicion [11] were given (in Swedish) on the form: *Obvious ACS,* Typical symptoms and ST-elevation with or without Q-waves on the ECG, or LBBB not known to be old; *Strong suspicion of ACS,* a.) Typical symptoms or b.) ST-T changes or LBBB not previously observed, or c.) Acute heart failure or hypotension regardless of ECG, or d.) Ventricular tachycardia or fibrillation or AV-block III; *Vague suspicion of ACS,* Unclear symptoms and history, non-ischemic ECG; *No suspicion of ACS,* No suspicion of ischemic heart disease or stable angina pectoris. These definitions where non-controversial and reflected common clinical reasoning at the hospital during the study.

The diagnostic criteria for ACS generally applied at the hospital during the study period were those of the European Society of Cardiology, the American College of Cardiology and the American Heart Association [21,22]. In the study patients, discharge diagnoses were made by the responsible ED physician, or, if the patient was admitted to inpatient care, by the responsible specialist ward physician.

### Statistical analysis

To get an overview of how the diagnostic tools were used to determine ACS suspicion, we present simple associations between the physician’s ACS suspicion on one hand, and TnT levels, ECG changes and symptoms on the other (Tables 1 and 2).

**Table 1:** The physician’s overall suspicion of ACS and the underlying assessments of the ECG, symptoms, and TnT

		**Assigned overall suspicion of ACS (%)**			
		**Obvious ACS**	**Suspicion**		
			**Strong**	**Vague**	**No**
*Total*		*1.8*	*21.7*	*38.1*	*38.3*
**ECG**	ST elevation, n = 24	70.8	8.3	8.3	12.5
	ST depression, n = 46	8.7	73.9	15.2	2.2
	T inversion, n = 35	0.0	74.3	20.0	5.7
	Ischemic ECG, n = 105	20.0	59.0	15.2	5.7
	Q-waves/LBBB, n = 13	0.0	38.5	38.5	23.1
	Normal ECG, n = 970	0.0	16.7	40.5	42.8
**Symptoms**	Typical of AMI, n = 147	11.6	59.2	29.3	0.0
	Typical of UA, n = 181	1.1	80.7	16.0	2.2
	Typical of ACS, n = 328	5.8	71.0	22.0	1.2
	Not specific for ACS, n = 408	0.5	3.7	80.4	15.4
	No suspicion of ACS, n = 415	0.0	0.5	9.4	90.1
**TnT**	TnT < 0.05, n = 1073	1.2	18.3	39.5	41.0
	TnT ≥ 0.05, n = 78	10.3	69.2	19.2	1.3

Ischemic ECG, ST elevation OR ST depression OR T inversion; Typical of ACS, Typical of AMI OR UA; LBBB, Left bundle branch block.

**Table 2:** Combinations of assessments of ECG findings, symptoms and TnT for cases with any suspicion of ACS

	**Assessments**			**Isolated assessments**			**Combinations of assessments**			
	**Ischemic ECG (ST elev or ST depr or T inv)**	**Symptoms typical of ACS (AMI or UA)**	**TnT+**	**Only ischemic ECG**	**Only symptoms typical of ACS**	**Only TnT+**	**Ischemic ECG + symptoms typical of ACS**	**Ischemic ECG + TnT+**	**Symptoms typical of ACS + TnT+**	**All 3**
*All patients, %*	*10.1*	*28.5*	*6.8*	*0.4*	*16.9*	*0.0*	*6.8*	*2.7*	*5.4*	*2.1*
Obvious ACS, n = 21	100	90.5	38.1	0.0	0.0	0.0	90.5	38.1	33.3	33.3
Strong suspicion, n = 250	26.8	93.2	21.6	0.0	51.6	0.0	22.4	7.6	20.4	6.8
Vague suspicion, n = 439	4.6	16.4	3.4	0.5	14.1	0.0	0.7	0.9	0.9	0.0

All figures percent. All three pathological = [ST elevation or ST depression or T inversion] and [typical symptoms of AMI or UA] and [positive TnT].

To further evaluate how the diagnostic tools simultaneously were used to determine the level of suspicion of ACS, two different logistic regression models were applied (Table 3). In the first model the binary response was any suspicion of ACS compared to no suspicion, while in the second model we evaluated obvious/strong suspicion of ACS compared to vague/no suspicion. ECG changes (4 categories; normal, ischemic, with LBBB or Q-wave, or with AF, AFL or pacemaker), symptom category, TnT-level (≥0,05 or < 0.05 μg/L), sex and dichotomized age (≥65 or < 65 years) were included as covariates in both models. The reference categories were normal ECG, symptoms raising no suspicion of ACS, TnT < 0.05, male sex and age < 65 years, respectively. Factors were considered significant if the P-value was below 0.05. Analyses were conducted with IBM SPSS Statistics 18 for Windows (IBM Corp., Somers NY, USA) software.

**Table 3:** Logistic regression analysis

		**Assignment of [obvious ACS] or [strong suspicion of ACS] or [vague suspicion of ACS] versus [no suspicion of ACS]**		**Assignment of [obvious ACS] or [strong suspicion of ACS] versus [vague suspicion of ACS] or [no suspicion of ACS]**	
		**P-value**	**Odds ratio (95% CI)**	**P-value**	**Odds ratio (95% CI)**
**ECG**	Ischemic ECG	0.127	2.68 (0.76-9.50)	< 0.001	30.6 (11.7-80.2)
	Q-wave or LBBB	0.154	4.38 (0.57-33.4)	0.027	11.1 (1.32-94.0)
	AF, AFL or PM	0.526	1.37 (0.52-3.60)	0.048	3.04 (1.01-9.15)
**Symptoms**	Typical of ACS	< 0.001	526 (185–1500)	< 0.001	620 (138–2780)
	Not specific for ACS	< 0.001	48.7 (31.6-75.1)	0.043	4.95 (1.05-23.3)
**TnT**	TNT+	0.112	6.55 (0.65-66.3)	0.007	3.35 (1.39-8.09)
	Age ≥ 65 years	0.001	2.16 (1.40-3.35)	0.014	1.90 (1.14-3.17)
	Female	0.913	1.02 (0.68-1.55)	0.043	0.59 (0.36-0.98)
	Intercept	< 0.001	0.074	< 0.001	0.003

Factors contributing to the overall assessment of the suspicion of ACS. Ischemic ECG = ST elevation or ST depression or T inversion; LBBB, Left bundle branch block; AF, Atrial fibrillation, AFL, Atrial flutter; PM, pacemaker; TnT+, TnT ≥ 0.05 μg/L.

## Results

As shown in Figure 1, out of 1222 consecutive chest pain patients, a total of 1151 patients were included in the study. Fifty-six patients were excluded because of incomplete study data. Six-hundred and twenty-one (54.0%) were hospitalized and 140 of those (22.5%) proved to have ACS as the discharge diagnosis. Characteristics for the included patients are given in Table 4. Mean age was 60.7 ± 18.5 (SD) years.

**Table 4:** Characteristics of the included patients

	**n**	**%**
*All patients*	*1151*	*100*
Women	505	43.9
Age ≥ 65 years	530	46.0
Angina Pectoris	248	21.5
Previous PCI/CABG	242	21.0
Diabetes Mellitus	168	14.6
Congestive heart failure	116	10.1
Atrial fibrillation	140	12.2
Previous stroke	103	8.9
Peripheral arterial disease	26	2.3
Use of warfarin	120	10.4
Use of statins	338	29.4

### Assessments of symptoms, ECG and TnT, and the overall likelihood of ACS

Table 1 shows the association between the designated likelihood of ACS and the underlying assessments of ECG, symptoms and TnT levels. Twenty-one (1.8%) of the 1151 patients were deemed as obvious ACS, 250 (21.7%) as strong suspicion of ACS, 439 (38.1%) as vague suspicion of ACS and 441 (38.3%) as no suspicion of ACS. Of the patients with ST-elevation, almost 71% were considered as obvious ACS. In contrast, only 5.8% of patients with typical symptoms of ACS were assessed as obvious ACS, and only 10.3% of those with a positive TnT. Instead, both patients with typical symptoms of ACS and patients with positive TnT were in some 70% of the cases assigned a strong suspicion of ACS. ST depression or T inversion in the ECG were typically associated with a strong overall suspicion of ACS (74% each). In patients with a normal ECG, ACS was still suspected in 57.2% of the cases. In contrast, when symptoms were non-suspicious of ACS, there was no overall suspicion of ACS in 90% of the cases.

There were associations between the assessments of the ECG, symptoms and the TnT level. Among patients with an ischemic ECG, 27.6% had a pathological initial TnT, compared to only 4.2% among those with a normal ECG. Similarly, TnT was positive in 18.9% of the patients with symptoms typical of ACS, compared to 0.5% in those with non-suspicious symptoms. Among patients with ST-elevation, 79.2% had symptoms typical of ACS. In patients with a normal ECG, a majority had symptoms not specific (36.6%) for, or not suspicious (39.9%) of, ACS.

### Combinations of assessments of symptoms, ECG, TnT versus overall likelihood of ACS

In Table 2 it can be seen that all patients assessed as obvious ACS had ischemic ECG changes, and that 90.5% had both ischemic ECG changes and symptoms typical of ACS. Among patients with a strong suspicion of ACS, typical symptoms were considered present in 93.2%. In patients with a vague suspicion of ACS, there was almost never any combination of assessments clearly indicative of ACS.

### Logistic regression analysis

In Table 3, the associations between the physicians’ level of ACS suspicion and covariates included in the logistic regression models are described. In the physicians’ assignment of any versus no suspicion of ACS, symptoms typical of and not specific for ACS contributed strongly and significantly (odds ratio OR 526 and 48.7, respectively, compared to no symptoms of ACS), but an ischemic ECG or a positive TnT did not.

In the assignment of an obvious/strong versus vague/no suspicion, symptoms typical of ACS was the most important factor (OR 620), but nonspecific symptoms (OR 4.95), ischemic ECG (OR 30.6) and a positive TnT (OR 3.35) also contributed significantly to this assessment. Since no patient with obvious ACS had a normal ECG or symptoms not suspicious of ACS, it was not possible to create a model to analyze the assignment of obvious ACS versus strong/vague/no suspicion.

## Discussion

The present results indicate that the ED physician uses the symptoms as the most important diagnostic tool when deciding the level of suspicion of ACS in chest pain patients, and that the ECG is considered more important than TnT. To the best of our knowledge, this study is the first to evaluate the relative importances of the symptoms, ECG and TnT in routine care. This study did not, however, analyze the optimal use of these diagnostic tools, i.e. their predictive values for the diagnosis of ACS. The results therefore do not show whether or not the physicians were correct in their use of the three different diagnostic modalities.

The predictive value of symptoms of ACS has been extensively studied, and although the symptom history is a cornerstone in the ED assessment, the limitations are well known [3-5]. No single symptom makes ACS highly likely or unlikely. For instance, the likelihood ratio (LR) for ACS/AMI of chest pain radiating to both arms or shoulders is only approximately 4–7, the LR of exertional chest pain 2.5, nausea and vomiting 2 and of positional chest pain 0.3 [6-9]. Some 30–50% of AMI patients lack chest pain [26], and among those with chest pain typical of AMI or ACS, 50% or less have it [10,11]. The chest pain quality, duration and severity are all suboptimal predictors of ACS [5,12,13]. Despite this, the ED physicians in the present study used the symptoms as the most important factor to determine the ACS likelihood. When ACS was ruled out, the symptoms provided the decisive information - neither the ECG nor TnT contributed significantly to the assignment of any versus no suspicion of ACS (Table 3). When symptoms were non-suspicious of ACS, the physician suspected ACS in less than one out of ten cases (Table 1). In addition, suspicions of ACS were sometimes based on symptoms alone, but almost never on ECG or TnT alone (Table 2).

When the physician could not rule out (i.e. assign no suspicion of) ACS, he or she also seemed to use symptoms as the most important diagnostic modality to grade the suspicion. In the regression model comparing obvious/strong with vague/no suspicion of ACS (Table 3), the odds ratio for symptoms typical of ACS was considerably higher than for ischemic ECG and positive TnT. Further, symptoms typical of ACS were clearly more often associated with a strong suspicion of ACS than were an ischemic ECG (Tables 1 and 2), and nonspecific symptoms were in >80% of the cases associated with a vague suspicion of ACS (Table 1).

The ECG has been considered to be the most valuable ED test in patients with possible ACS, providing almost as much information as all other information combined [4,5]. This view is supported by published statistical decision support models, where ECG data have invariably been found to be crucial for the prediction of ACS in the ED, as opposed to data on symptoms and blood markers of myocardial injury [27]. In some models with ECG variables only, adding symptoms and other clinical variables did not improve ACS prediction [28]. In the present study, the ECG was indeed the most important factor when the ED physicians identified a case of obvious ACS (Tables 1 and 2), i.e. when ACS was ruled in. However, the ECG was not considered as valuable for grading the ACS suspicion, and for ruling out ACS. A majority of patients with a normal ECG were still suspected to have ACS (Table 1), and the ECG did not contribute significantly to the assessment of any versus no suspicion of ACS (Table 3). A possible cause of this is that the shortcomings of the ECG for ACS prediction were recognized by the physicians in this study. Some 20% of AMI patients and 40% of UA patients have normal ECGs in the ED [14]. Only about one in four of those with significant ST depression prove to have ACS [15], and only 5% with T-wave changes meeting ACS criteria have AMI [16]. Further, the ECG often does not detect transient myocardial ischemia [17], ischemia in patients with prior AMI [18], or ischemia in the area of the left circumflex coronary artery [19]. These limitations may be even more clinically relevant in EDs with a prehospital ECG system, such as in Lund, where patients with marked and clear-cut ECG changes (i.e. ST elevation myocardial infarction) usually bypass the ED on the way to the angiography suite.

Perhaps as expected, TnT was the least valuable diagnostic tool to the ED physicians. TnT was not a significant factor in the assignment of any versus no suspicion of ACS, and had a markedly lower odds ratio than ischemic ECG and typical symptoms in the assessment of obvious/strong versus vague/no suspicion of ACS (Table 3). In six patients out of ten with a normal TnT, the physician still suspected ACS (Table 1), and in only 10% of the patients with a positive TnT, the physician noted an obvious ACS. TnT’s small role for the ACS suspicion was probably due to its limited sensitivity and specificity for ACS in the ED [29], and it remains to been seen if newer high-sensitivity assays [30-32] will increase the importance of TnT in the assessment of patients with a possible ACS.

Efforts to improve ED decisions are best based on an understanding of the practical decision-making in routine care. Although the ECG might theoretically be superior to symptoms when predicting ACS, it may not be surprising to the practicing ED physician that symptoms emerged as a more important method to decide ACS suspicion than ECG and TnT in this study. The patient’s description of his or her symptoms includes a multitude of information (ranging from the pain localization to concurrent symptoms and perhaps even the clarity of the description) that physicians integrate when assessing the patient. Much of this information is difficult to quantify and study with traditional research protocols, and hence also to include in decision support models. Further, combinations of symptoms are very common and are even more difficult to study. We therefore believe that the practical importance of symptoms for ACS prediction, and especially the combination of symptoms, is larger in routine care than suggested by published studies on predictive values [6-9]. Further investigation of ACS prediction based on symptoms is needed, and also of the incorporation of symptom information in decision support models. For the time being, optimal decision-making in cases of possible ACS may involve physician interpretation of the symptoms and computerized ECG interpretation, since modern computer models are generally superior to physicians in detecting ACS on the ECG alone [33-35].

### Limitations of the study

This study was performed at one university hospital ED only, and the results are not necessarily generalizable to other physician or patient populations. Definitions of the different levels of overall suspicion of ACS were given on the study forms. Although these definitions were non-controversial and did not specify which diagnostic modality is the most important, they most likely influenced the physicians’ assessments. Different definitions (or no definitions) may therefore have led to somewhat different results. Changing assessment scales for the symptoms and the overall likelihood of ACS might also have changed the results.

The study was designed so that the physician’s interpretations of the ECG, symptoms and TnT were not isolated from each other. The physician was thus aware of the ECG when assessing the symptoms and vice versa. This is not always the case in routine care, and our results may therefore not be applicable to the ED physician’s clinical reasoning in each individual patient case. Larger studies at other centers are needed to confirm our findings, perhaps also with different definitions of the levels of ACS suspicion.

## Conclusion

Although the ECG may theoretically be the most important diagnostic tool in chest pain patients with possible ACS, the present results indicate that ED physicians do not use the ECG in this way. Instead, the physicians used symptoms as the most important assessment tool, and applied primarily the symptoms to determine the level of suspicion of ACS and to rule out ACS. The ECG was only primarily used to rule in ACS, whereas the TnT level in general played a minor role for the ACS likelihood. To our knowledge, this study is the first to evaluate the relative importances of these diagnostic tools in routine care. Further studies of ACS prediction based on symptoms may help improve ED decision-making in patients with possible ACS.

## Competing interests

The authors declare that they have no competing interests.

## Authors’ contributions

AK analyzed and interpreted the data, wrote and critically revised the manuscript. MS collected and interpreted the data and critically revised the manuscript. UE conceived and designed the study, interpreted the data and wrote and critically revised the manuscript. All authors read and approved the final version of the manuscript.

## Pre-publication history

The pre-publication history for this paper can be accessed here:

http://www.biomedcentral.com/1471-227X/14/9/prepub
